# Are higher unintended pregnancy rates among minorities a result of disparate access to contraception?

**DOI:** 10.1186/s40834-020-00118-5

**Published:** 2020-10-01

**Authors:** Michele Troutman, Saima Rafique, Torie Comeaux Plowden

**Affiliations:** 1 Department of Obstetrics and Gynecology, Beth Israel Deaconess Medical Center, Boston, MA USA; 2 Advantia Ob/Gyn Shady Grove, Rockville, MD USA; 3 Department of Obstetrics and Gynecology, Womack Army Medical Center, Ft Bragg, NC USA

## Abstract

**Abstract:**

Unintended pregnancy is a major global issue. Women who experience an unintended pregnancy have a significant risk of morbidity and mortality. Additionally, these women also experience substantial financial hardships. Many women, particularly women of color, do not have adequate access to reliable and affordable contraception resulting in major health disparities among this group. This review explores the relationship between unintended pregnancy and inadequate access to contraception and is divided into 5 sections: addressing problems associated with unintended pregnancies, unintended pregnancy rate in the US, disparities of unintended pregnancy rates and access to care, addressing potential solutions, and finally conclusions.

**Keyterms:**

unintended pregnancy, healthcare disparities, contraception, access to care.

## Introduction

An unintended pregnancy is any unplanned, mistimed or unwanted pregnancy at the time of conception [1]. In 2011, 48% of all pregnancies in the United States were unintended [2]. Similarly, women worldwide have high unintended pregnancy rates. In 2010–2014, 44% of all pregnancies worldwide were unintended [3]. Although we’ve begun to see a slow decline in these numbers with the aid of education, LARC methods, and access to family planning services, unintended pregnancy rates remain a major public health problem.

Unintended pregnancies have a substantial impact on public health. Women with unintended pregnancies have a higher percentage of late entry to care, alcohol and drugs use during pregnancy and higher rates of preterm birth [4]. Unintended pregnancies are often higher among adolescents, lower income, minority, and single women who have poverty rates twice that of other groups, making the financial impact of an unplanned conception even greater [5]. Improved access to contraception by age 20 has been shown to decrease the likelihood that a woman will subsequently live in poverty and thus increase one’s quality of life [6]. With the onset of coronavirus disease 2019 (COVID-19) and the resurface of a national economic depression, it is important that now more than ever we consider the economic burden of these unintended pregnancies and the strain on national resources. Many organizations are significantly decreasing in-person and telehealth visits and reproductive access organizations have been forced to innovate in a more thoughtful way to reach those most at risk. There are slated state and national healthcare budget cuts although it remains unclear the long-term effects it will have on unintended pregnancies. While it is possible to increase clinic show rates with the use of technology and decreasing economic barriers, what remains is the constant gaping disparities in digital equity and patient perception regarding the care, or lack thereof, that they’re receiving.

Unintended pregnancy is a public health emergency. The U.S. Department of Health and Human Services has identified reducing unintended pregnancy as a significant goal in the Healthy People 2020 family planning objectives. The initiative focuses on improving pregnancy planning, spacing and prevention as a way to improve health outcomes while decreasing the economic burden. In order to accomplish the goal, barriers such as limited access to publicly funded services, limited transportation, lack of youth-friendly services, and inadequate services for men are identified as some of the issues to be addressed [7].

Although women of reproductive age of all races and ethnicities are at risk of unintended pregnancy, Hispanic and Black women are disproportionally at risk [8]. Globally, the at-risk population numbers are in the millions, making this a critical issue worldwide. Contributions to this disparity include income, insurance status, relationship status, and education level. Our objective was to examine the contributors to high rates of unintended pregnancies and to identify potential strategies to address this critical issue.

## Unintended pregnancy rates in America

**Fig. 1:**
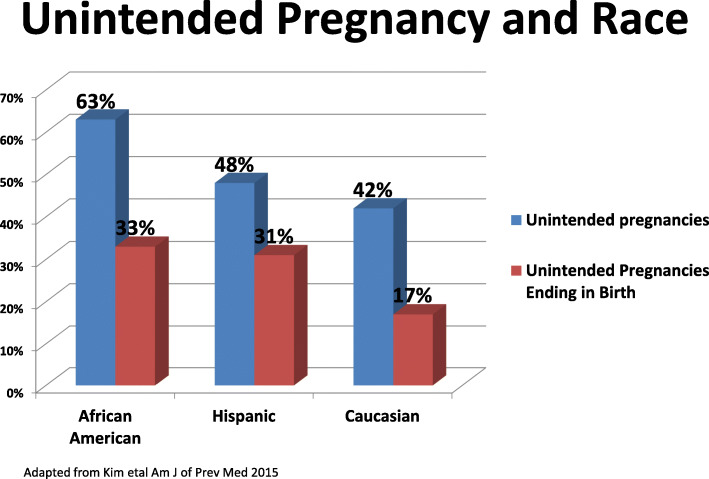
Unintended pregnancy and race

In 2011, the rates of unintended pregnancy among African-American, Hispanic and Caucasian women that ended in birth were 33, 31, and 17% respectively in the United States [2]. Using data from the National Survey of Family Growth from 2006 to 2010, Kim et al. observed 50% of the 3577 pregnancies were unintended [9]. The study notes that women of color, particularly black women had higher rates than whites; 63% (Black women), 48% (Hispanic women) and 42% (Caucasian women). Although the rates were higher among black and Hispanic women, the difference was not statistically significant (Fig. 1). However, even the lowest rate of unintended pregnancy, 42%, would be considered unacceptable high by most healthcare providers. This number is particularly alarming given the failure rate of less than 1% for highly effective contraceptives [10].

## Disparities in unintended pregnancy and access to care

The medical literature has documented racial and ethnic disparities in access to healthcare, as well the quality of that care for more than a decade. In 2003, the Institute of Medicine determined “research suggests that healthcare providers’ diagnostic and treatment decisions, as well as their feelings about patients, are influenced by patients’ race or ethnicity and that these differences may contribute to disparities in health outcomes” [11]. Multiple studies have unfortunately demonstrated that African-American and Latina women from low socioeconomic backgrounds have been strongly encouraged to limit their family sizes and have been pressured to start contraception or proceed with tubal sterilization [12, 13].

Historically, the United States has had a shameful history regarding forced sterilizations and reproductive injustices which may lead minority women to be distrustful of contraception [14, 15]. There are also racial differences in contraceptive preferences. Because it is important to many African American women to avoid the use of hormones, they may be less likely to choose highly effective contraception such as implants, injectable contraception or levonorgestrel containing intrauterine devices (IUDs) [15]. Outside of preferences guided by historical weight, there are also distinctive barriers to reproductive access.

This idea of disparity in contraceptive access is echoed in the discordance between desired fertility and chosen method of contraception. A study of 110 (48 African-Americans, 43 Hispanics, 19 Caucasians) low-income women who lived in an underserved area found that 40% of women who did not desire pregnancy had unprotected intercourse within the last 12 months [16]. Similarly, a study examining contraception trends found that 16% of African-American women who were at risk of unintended pregnancy were not using contraception compared to about 9% of Hispanic, white and Asian women [8].

Attitudes and norms regarding contraception in minority groups are often different than those of Caucasian women. Frequently, when African-American and Latina women choose contraception, they choose less effective contraception options (i.e. condoms) compared to white women [12–14]. Interestingly, data from the contraceptive CHOICE project revealed that prior to enrollment in the study, African-American women who have had a history of discrimination were more likely to choose less efficacious contraceptive measures (specifically barrier methods, natural family planning and withdrawal) but after enrollment, 67% of these women elected to use long-acting reversible contraception (LARC) [16]. The script that was used in the CHOICE study provided important information about the effectiveness of various contraceptive methods, and patients were free to choose whatever method they desired. Patient education played a pivotal role in the success of the CHOICE project, but it is imperative that other contraceptive programs provide education and not coercion.

High unintended pregnancy rates, particularly among low-income women and women of color, have persisted despite the expanded options for highly efficacious contraceptives. LARC, which includes IUDs and subdermal contraceptive implants, are cost-effective and highly efficacious, with an annual failure rate of 0.05% (implants) and 0.2–0.8% (IUDs) [17]. Despite the efficacy of these methods, they are often associated with up-front out-of-pocket costs which may be prohibitive to poorer women [10, 17]. When women were given multiple options for contraception, and cost was not a consideration, 67% elected to use long-acting reversible contraceptive (LARC) [18]. In comparison, non-LARC contraceptive options were 20 times more likely to have an unintended pregnancy, demonstrating how much effective contraception is of paramount importance for preventing unintended pregnancy [19].

Any efforts to decrease unintended pregnancy will need to include elimination of barriers such as a lack of insurance, inadequate coverage that requires large out of pocket expenses, or extremely high premiums. One potential strategy is to educate third party payers about the cost savings associated with widely available contraception. One study estimated that the direct medical cost of unintended pregnancy in the United States was more than $4.6 billion annually [20]. Women ages 18–24 have the highest risk of unintended pregnancy. Using cost models, one review found that if 10% of women ages 20–29 who used oral contraception changed to LARC, the total costs associated with unintended pregnancy would decrease by $288 million per year [20]. Third party payers should consider access to contraception as effective preventive health care that will decrease medical costs.

Although decisions regarding contraception are often left up to the female partner, the role of the male partner must be examined as part of the strategy to decrease unintended pregnancy. Utilizing data from the 2006–2010 National Survey of Family Growth, one study attempted to better understand knowledge of contraception among young men [21]. Researchers found that although 96.6% of men reported formal sex education, Black men were less likely to receive contraceptive education [21]. Another study examining contraceptive knowledge found that men were more likely to “display serious gaps in objective knowledge about the major contraceptive options” [22]. Despite men often being left out of conversations involving contraception, counseling that reflects a couple’s culture and values may help increase compliance especially amongst minority groups [23].

Age plays a significant role in the likelihood of having an unintended pregnancy. Young women who become sexually active at an early age are particularly at risk for an unplanned conception. Data from Demographic and Health Surveys revealed that a significant proportion of adolescents in 16 countries reported sexual activity. In 9 of these 16 countries, approximately 40% of women reported sexual activity before age 18 while men engaging in intercourse before age 18 ranged from 25 to 75% [24]. There is a clear need for access to contraception among adolescents in many of these countries. However, adolescents in low and middle-income countries face additional barriers regarding contraception. These include poor understanding of how to use various methods correctly and law or policies preventing young unmarried women from accessing contraception [24]. The following recommendations are potential solutions to the disparities of contraception access facing minority population.

## Addressing the problem: potential solutions [Table 1]

The Centers for Disease Control (CDC) recommends that “every woman, man and couple should be encouraged to have a reproductive life plan” [25].

**Table 1:**
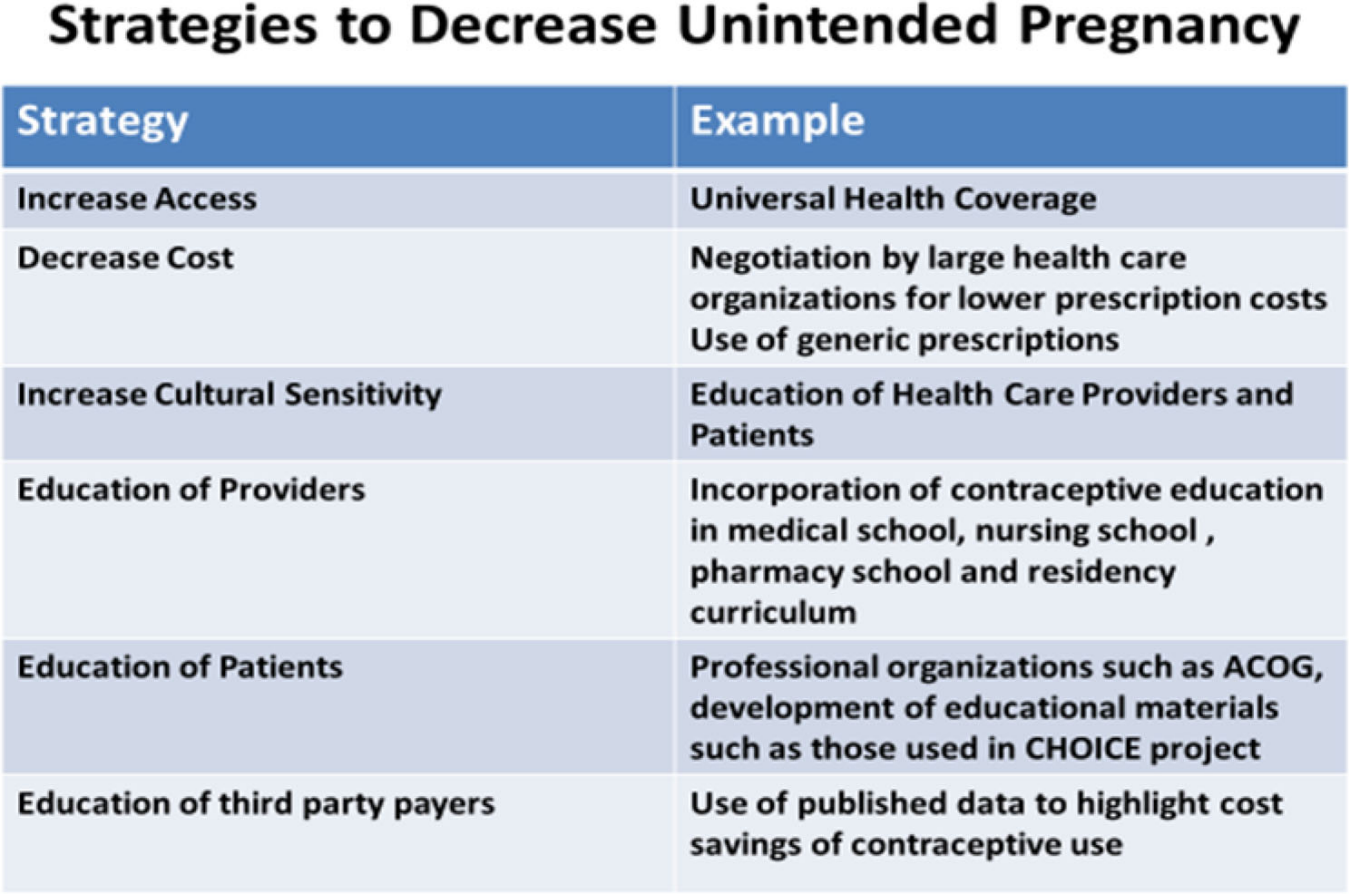
Strategies to Decrease Unintended Pregnancy

### Access/cost

Although multiple factors affect the unintended pregnancy rate, access to reliable and safe contraception can certainly positively impact this problem. Universal coverage of all contraceptive methods has been shown to decrease unintended pregnancy rates [12]. Negotiation with pharmaceutical companies by large health care organizations, such as HMOs, is also a potential strategy to decrease cost. On a global level, organizations like the Gates Foundation are investing in reasons for non-use, distribution and development for contraceptive technology [26].

### Culturally sensitive approach

African-American and Latina women suffer from misconceptions surrounding contraception, its use and efficacy [14]. The CHOICE project serves an example of using patient education to effectively overcome cultural beliefs that pose a barrier to the use of highly efficacious contraception. Access to care limitations based upon race, ethnicity and income is well documented and poses one of the most significant impediments to contraceptive access. Incorporation of contraceptive education as well as culturally sensitivity training into the curriculum of medical, nursing, and pharmacy schools and residency training programs are a tool that can be used to address these important issues.

### Appropriate physician education

Like patients, health care professionals often have limited knowledge about the availability and appropriate use of contraceptive methods. IUD use, one type of LARC, has been hampered by the persistence of incorrect myths including that they are contraindicated in young/adolescent nulliparous women, they lead to infertility and women will experience high rates of infection [17]. Residency programs in obstetrics/gynecology, family practice and pediatrics along with nurse practitioners and physician assistants should ensure that providers are aware and well-educated about LARC methods and its contraindications. Appropriate counseling by providers “not only promotes effective methods but also directly translates to increased use” [14]. Use of published materials by organizations such as the American College of Obstetricians and Gynecologists (ACOG), Society of Family Planning (SFP), and the use of apps such as Medical Eligibility Criteria app produced by the CDC (https://www.cdc.gov/reproductivehealth/contraception/usmec.htm) facilitate the appropriate selection of contraceptives for women with medical conditions such as hypertension. These tools aid women in locating effective contraception that they otherwise could have been denied as a result of a provider’s fears about medical complications.

### Maximizing opportunities to educate patients

The ACOG strongly advocates for reproductive planning. In a recent committee opinion, the College emphasized that physicians should take every contact with their patients as a teachable moment [27]. Rather than limiting the discussion of contraceptive management to well women or contraceptive visits, every visit should be considered as a unique opportunity to address patient’s reproductive plan and offer counseling, education and correct any misconceptions on available contraceptive options. Initiatives like “One Key Question” and “Providing Quality Family Planning Services” aim to enhance effective communication and improve understanding of a woman’s reproductive health plan [28]. Adopting these strategies in day-to-day interactions will efficiently utilize available resources and further achieve the goal of preventing unintended pregnancies [29].

### Empowering other health care providers

Emergency contraceptives is another excellent option geared towards decreasing the unintended pregnancy rates [30]. Now that these methods are available over the counter, many patients may seek advice from pharmacists [31]. Pharmacists can advise patients of emergency contraception, but, additionally can provide information regarding LARC and refer to an appropriate provider [31]. Nurse practitioners are becoming the primary providers of contraception for some women. Training these healthcare providers to provide not only counseling about LARC, but also placement of IUD and implants will help women access these methods more easily [31].

### Using community resources

Disseminating information through various avenues including schools, health centers, the media, and peer education has been shown to improve uptake of LARC use [31]. Additionally, other resources that should be considered when targeting minority groups may include churches, community center, and salons. One limitation of LARC use is a lack of appropriately trained personnel. Charyeva et al. in their study in Northern Nigeria showed increased utilization of LARCS by training community health extension workers in their insertion and removal [32]. In Ethiopia, similar results were noted in the utilization of implants with the help of health extension workers [33]. These studies suggest that optimizing human resources and training ancillary staff to deliver these services could be another option in increasing the utilization of contraceptives and eventually decreasing unintended pregnancies in at risk communities worldwide.

### Title X and reproductive access

Title X is a federal grant program through the US Department of Health and Human Services that provides comprehensive family planning and preventative health services, prioritizing low income families [34]. Recent proposed revisions to Title X will negatively impact access to legal abortion services and impede the provider’s ability to discuss family planning options at federally funded centers. The regulations would most affect low income and minority women. Organizations like Planned Parenthood who serve 41% of Title X recipients, use funding to provide preventative services and prevent 1 million unintended pregnancies a year [35]. If these regulations are enacted, low income and minority women who rely on Title X centers for their reproductive needs will be much more vulnerable to unintended pregnancies.

## Conclusion

Unintended pregnancy continues to be a critical global issue, particularly among certain ethnic and racial groups as well as low income women. These unintended pregnancies can negatively impact women physically, emotionally, and financially. Easier access to effective contraception methods, particularly long-acting reversible contraception, can certainly help to address this public health issue. Physicians and other health-care providers need to ensure they are providing comprehensive care and have received appropriate contraceptive education as well as culturally sensitive training. Although access to contraception plays a large role in unintended pregnancy, the added impact of less efficacious contraceptive methods use, lack of patient and provider education and understanding are additional contributors. Contraceptive programs must utilize multiple wide ranging strategies to achieve success in decreasing unintended pregnancies and its surrounding disparities.

## Data Availability

Not applicable.

## Abbreviations

COVID-19: Coronavirus disease 2019
IUD: Intrauterine device
LARC: Long acting reversible contraception
CDC: Centers for disease control
ACOG: American college of obstetricians and gynecologists
SFP: Society of family planning
